# A Polymer Physics Investigation of the Architecture of the Murine Orthologue of the *7q11.23* Human Locus

**DOI:** 10.3389/fnins.2017.00559

**Published:** 2017-10-10

**Authors:** Andrea M. Chiariello, Andrea Esposito, Carlo Annunziatella, Simona Bianco, Luca Fiorillo, Antonella Prisco, Mario Nicodemi

**Affiliations:** ^1^Dipartimento di Fisica, Università di Napoli Federico II, Naples, Italy; ^2^Istituto Nazionale Di Fisica Nucleare Napoli (INFN), Complesso Universitario di Monte Sant'Angelo, Naples, Italy; ^3^Max Delbrück Center for Molecular Medicine in the Helmholtz Association (MDC), Berlin, Germany; ^4^Institute of Genetics and Biophysics, Consiglio Nazionale Delle Ricerche (CNR), Naples, Italy; ^5^Berlin Institute of Health, Berlin, Germany

**Keywords:** polymer physics, chromatin, Neuroscience, congenital disease, *7q11.23* locus

## Abstract

In the last decade, the developments of novel technologies, such as Hi-C or GAM methods, allowed to discover that chromosomes in the nucleus of mammalian cells have a complex spatial organization, encompassing the functional contacts between genes and regulators. In this work, we review recent progresses in chromosome modeling based on polymer physics to understand chromatin structure and folding mechanisms. As an example, we derive in mouse embryonic stem cells the full 3D structure of the *Bmp7* locus, a genomic region that plays a key role in osteoblastic differentiation. Next, as an application to Neuroscience, we present the first 3D model for the mouse orthologoue of the Williams–Beuren syndrome *7q11.23* human locus. Deletions and duplications of the *7q11.23* region generate neurodevelopmental disorders with multi-system involvement and variable expressivity, and with autism. Understanding the impact of such mutations on the rewiring of the interactions of genes and regulators could be a new key to make sense of their related diseases, with potential applications in biomedicine.

## Introduction

Novel and different technologies, such as Hi-C (Lieberman-Aiden et al., 2009) and GAM (Beagrie et al., 2017), are revealing that, in higher organisms, chromatin is folded in the nucleus of cells with complex 3D spatial organization (Lanctôt et al., 2007; Misteli, 2007; Bickmore and van Steensel, 2013; Tanay and Cavalli, 2013; Dekker and Mirny, 2016). Chromosomes are organized into arrays of megabase-sized topologically associating domains (TADs), characterized by strong local interactions (Dixon et al., 2012; Nora et al., 2012; Beagrie et al., 2017); TADs, in turn, can interact between each other generating higher order structure, called meta-TADs, extending across genomic scales (Fraser et al., 2015); and patterns are seen also within TADS (Sexton et al., 2012; Phillips-Cremins et al., 2013). The 3D structure of chromatin has crucial functional roles since, for instance, gene activity can be controlled by chromosomal interactions through the formation of long-range contactas between gene and their regulators. Nevertheless, the mechanisms regulating chromosomes architecture are only partially understood.

In order to elucidate the genome-wide contact data and to identify the mechanism underlying chromatin 3D organization, quantitative models, based on principles of polymer physics, have been developed in the last few years (reviewed, e.g., in Nicodemi and Pombo, 2014). Some of them focus on the molecular mechanisms driving chromatin folding, such as the interactions with binding molecules (Nicodemi and Prisco, 2009; Barbieri et al., 2012; Brackley et al., 2013; Jost et al., 2014; Chiariello et al., 2016); some others consider dynamic processes, involving the transient formation of loops (Bohn and Heermann, 2010) or mechanisms where the polymer is extruded dynamically by specific extruding molecular factors (Sanborn et al., 2015; Fudenberg et al., 2016). In others entire chromosomes are modeled, based topological and kinetic constraints (Rosa and Everaers, 2008; Di Stefano et al., 2016). Here we focus on the String&Binders Switch (SBS) model, which appears to be able to explain in a quantitative way Hi-C, GAM and FISH data within a single framework (Nicodemi and Prisco, 2009; Barbieri et al., 2012; Fraser et al., 2015; Annunziatella et al., 2016; Chiariello et al., 2016; Beagrie et al., 2017). In the SBS model specific interactions between DNA-binding molecules determine the formation of non-random chromatin loops (Barbieri et al., 2012; Annunziatella et al., 2016; Chiariello et al., 2016). Within the SBS model, contact probabilities from many independent Hi-C datasets (Annunziatella et al., 2016; Chiariello et al., 2016) can be explained with a high degree of accuracy. Here we review the basic underlying concepts of polymer physics and, in particular, we report our results about the 3D structure of *Bmp7* locus (Chiariello et al., 2016) in mouse empbyonic stem cells (ESC-46C). As an application of potential interest to neurogenetics, we reconstruct for the first time the three dimensional structure of the *7q11.23* locus, where structural variants are associated with severe disorders such as autism and Williams-Beuren syndrome (e.g., Sanders et al., 2011 and ref.s therein).

## Polymer models of chromatin organization

**Figure 1:**
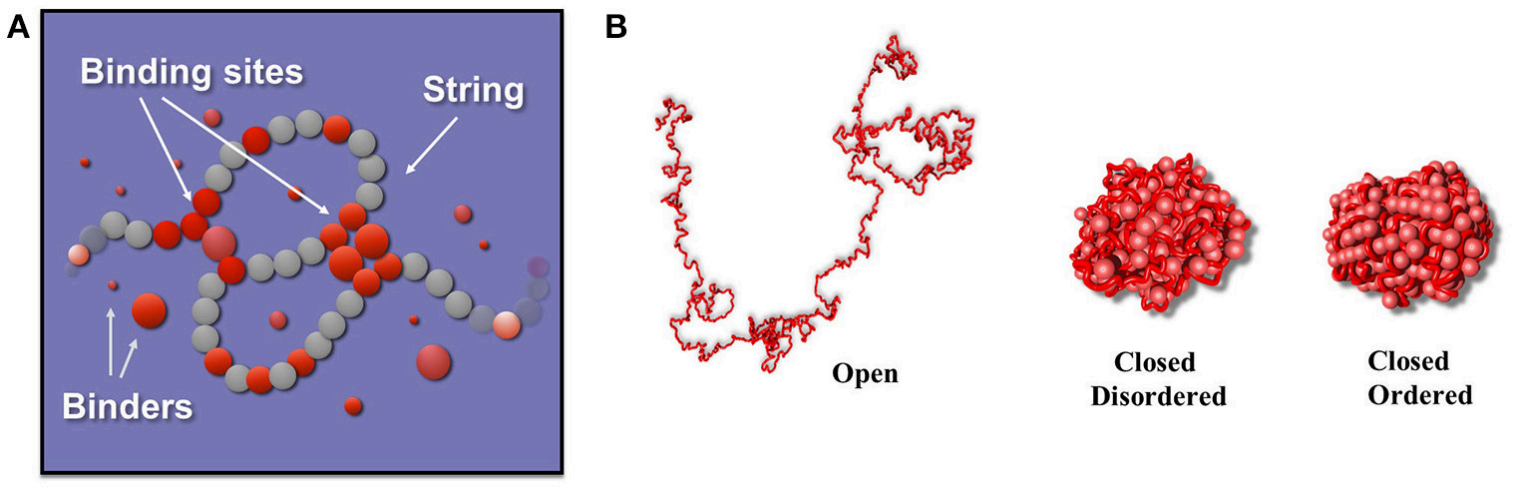
The Strings and Binders model of chromatin. **(A)** The StringandBinders (SBS) model aims to quantify the scenario where chromatin folding is established by diffusing molecular factors such as Transcription Factors. The figure shows a toy version of the model including only one type of binders (red spheres): *c* is the binder concentration and *E*_*X*_ their binding affinity. **(B)** Such a simple toy model has three main stable conformational classes (images adapted from Chiariello et al., 2016): a *coil* state where the polymer conformation is an open Self-Avoiding Walk (SAW) chain randomly folded (polymer on the left); a disordered *globule* state where the polymer is closed in a compact, disordered conformation (center) and an ordered *globule* state where the polymer and the binders are in an ordered, crystalline-like, conformation (right).

In the SBS model a chromatin filament is represented as a Self-Avoiding Walk (SAW) polymer made of consecutive beads which can interact with diffusing particles (binders, Figure 1A). The interaction between binders and bead chain spontaneously gives rise to the typical loops that are present in the genome. In the simplest version, only one type of binders are present, and can interact with all the beads of the chain, homopolymer model (Nicodemi and Prisco, 2009; Barbieri et al., 2012, 2013; Annunziatella et al., 2016; Chiariello et al., 2016). The binding-molecules have a concentration *c* and a binding affinity *E*_*X*_ By tuning the control parameters *c* and *E*_*X*_ the system folds in its different stable conformational states, as dictated by polymer physics. It is possible to show that at least three stable states exist (Annunziatella et al., 2016; Chiariello et al., 2016; Bianco et al., 2017) the coil state, observed for low values of affinities *E*_*X*_ and concentrations *c*, where the chain behaves as a SAW polymer because of binder-beads loops are unstable; the globular state, where the interaction is strong enough to fold the polymer in a compact conformation. In this phase the binders can form a disordered structure or, for higher *c* and, especially, *E*_*X*_ values, an ordered structure (crystalline-like) although they have not any direct interaction with each other (Chiariello et al., 2016; Bianco et al., 2017). Different polymer conformations in the same class are thermodynamically indistinguishable. In Figure 1B three exemplificative structures are shown, one for each stable class.

## Chromatin as a mixture of differently folded regions

**Figure 2:**
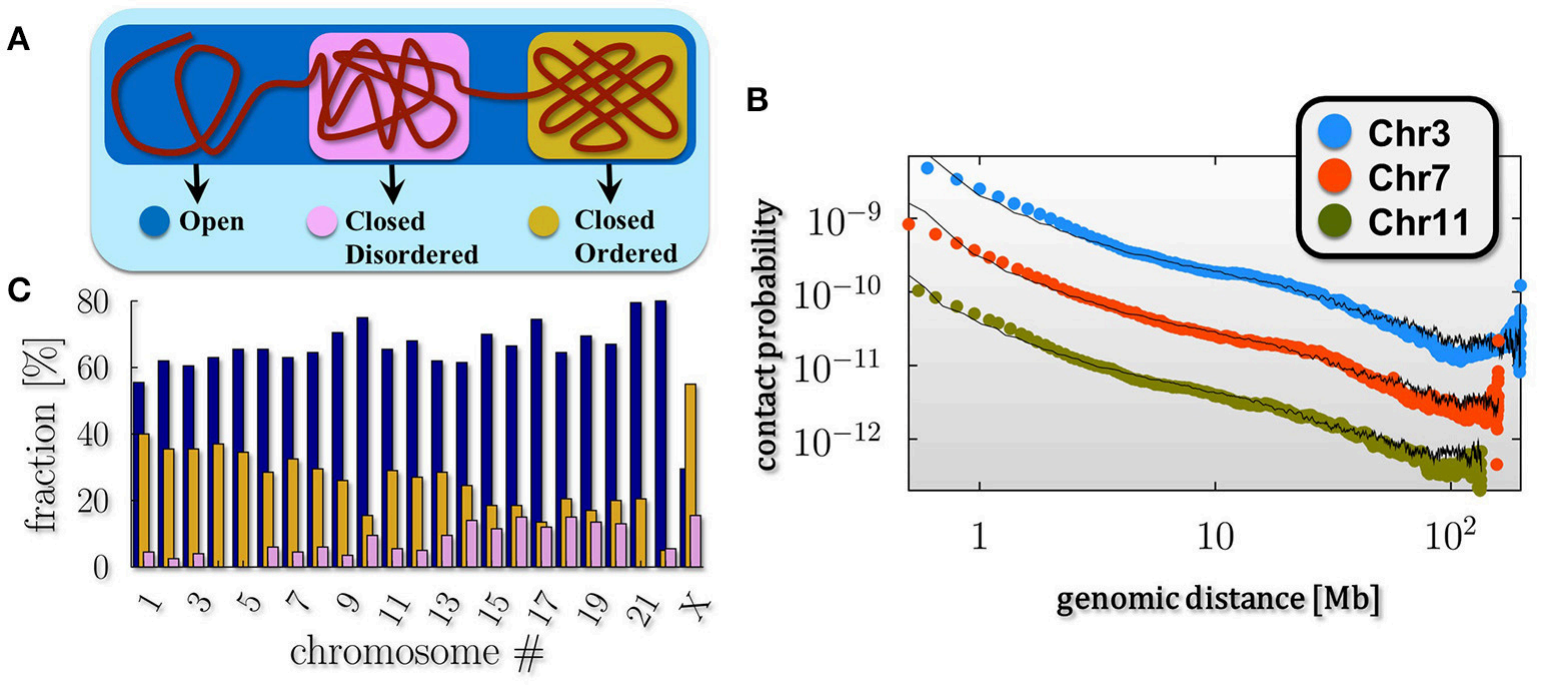
Chromosomes are a mixture of differently folded regions. **(A)** The polymer mixture model of chromatin poses that a chromosome is a mixture of differently folded region, each belonging to the stable folding classes described by polymer physics (pure states), as discussed in Figure 1. In this scenario the average pairwise contact frequency is determined only by the relative abundances of the pure states in the mixture. **(B)** Within such a model, the decay of the average pairwise contact data as a function of genomic separation of single chromosomes from in-situ Hi-C data in fibroblast (IMR90) cells (data from Rao et al., 2014) can be fitted over three orders of magnitudes in genomic separation. **(C)** The barplot shows the relative composition of the pure states across the different chromosomes for the IMR90 cell line: chromosome richer in genes, such as chr17 or chr19, result to be composed, for the most part, by open regions (up to 70%), while chromosome X, as expected, has a more compact conformation compared to the other chromosome, with percentage of closed state above the 60%.

The thermodynamic classes just described represent the stable conformations of the polymer. It is well established from microscopy and Hi-C experiments that chromosomes are typically organized in eu- and heterochromatin, that is genome regions where chromatin is in an more open and in a more compact state respectively. This consideration points toward a simple, yet approximated picture of chromatin where single chromosomes are mixtures of differently folded regions, belonging to the stable classes (*pure states*) predicted by polymer physics (Annunziatella et al., 2016; Chiariello et al., 2016), as schematically depicted in Figure 2A.

To test this *pure state mixture* model of chromatin we compare the experimental average pairwise contact probability *P(s)* of two generic loci separated by a genomic distance *s* with the theoretical *P(s)*. In this scenario, the average *P(s)* results simply from a linear combination of the contact probabilities in the three pure state regimes. Thus, the theoretical *P(s)* depends only on the fractions of these states in the combination, which are best fit parameters (Barbieri et al., 2012; Chiariello et al., 2016). In Figure 2B we show the results of this approach from the analysis of another recent published Hi-C dataset in different chromosomes, from fibroblast (IMR90) cells (Rao et al., 2014). Superimposed in black are the corresponding mixture model fits, which highly match the experimental behavior (chi-squared test across all chromosomes ranging from 6 × 10^−3^ to 2.5 × 10^−1^). This analysis shows that this simple model quantitatively explains the average genome-wide or single chromosome contact data for a wide range of genomic lengths, from 0.5 Mb up to chromosomal scale, as found previously for other analyzed datasets (Chiariello et al., 2016).

The mixture composition that best describes the experimental data for the chosen cell line is estimated (Figure 2C; errors below 10% of signal, not shown in the plot). It results to be strongly dependent on the considered chromosome: for instance, chromosome X is composed by mostly closed regions (above the 60%) with both the closed states (ordered and disordered) present (in a ratio about 3/2) while chromosomes richer in genes, such as chromosome 17 or 19, are up to 70% open in IMR90 cells. Briefly, the open-close combination reflects the distribution of differently folded domains along the chromosomes, across their thermodynamics states as also recently confirmed by the completely different experimental technique GAM (Beagrie et al., 2017).

## Topological domains and higher-order structures

**Figure 3:**
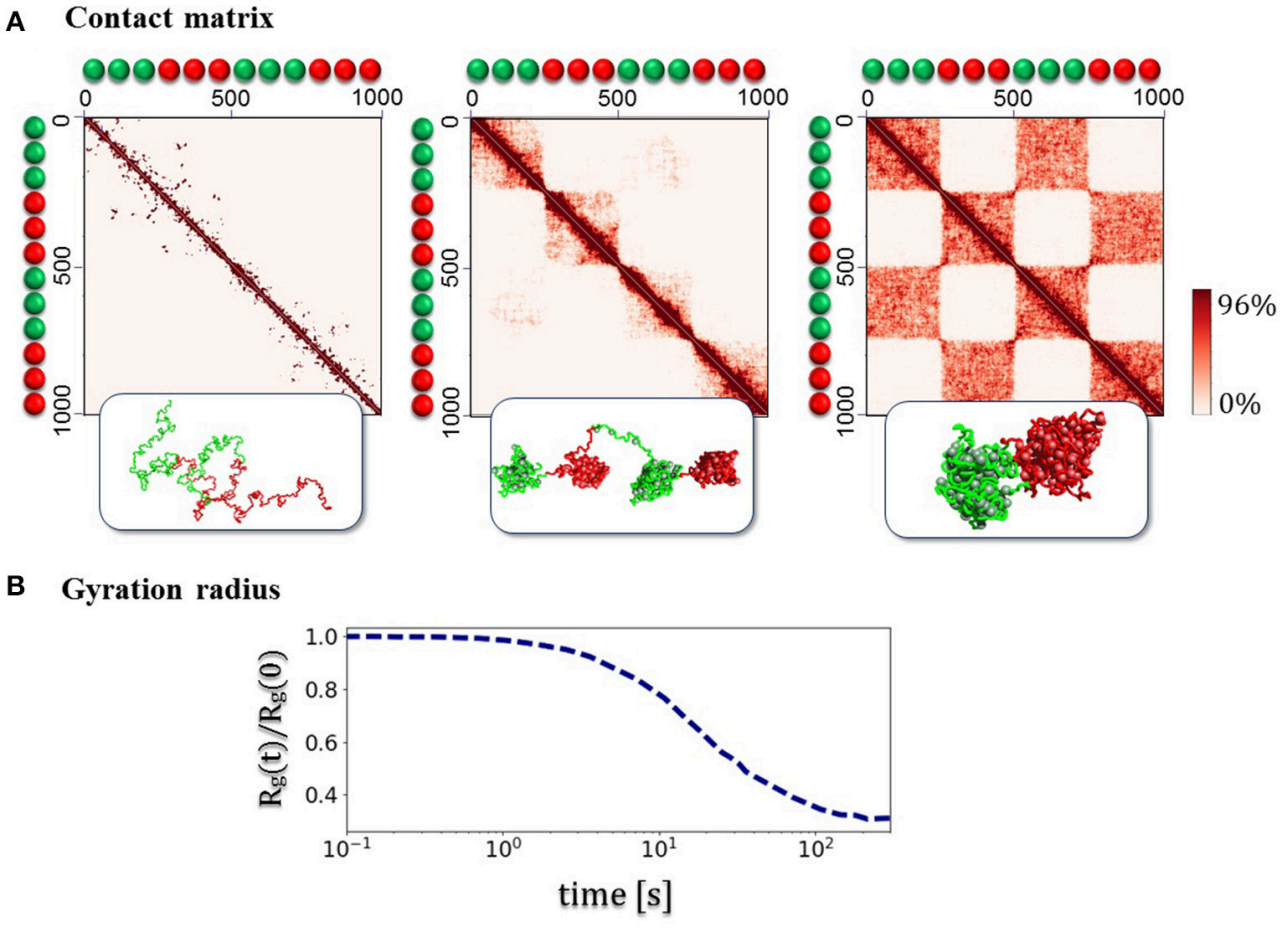
Assembly of topological domains and higher-order structures. **(A)** The figures illustrates the folding dynamics of a block-copolymer toy model where only blocks of two different binding sites (green/red) are present, with blocks alternated along the polymer. The system spontaneously folds in a compact state where initially local separated domains (as the TADs in the Hi-C data, Dixon et al., 2012; Nora et al., 2012) are formed, and at equilibrium a hierarchy of higher-order domains appears (as the metaTADs in the Hi-C data, Fraser et al., 2015). **(B)** The folding dynamics of the system from an initial SAW conformation is marked by the decrease of the gyration radius *Rg(t)* as a function of time *t*. In the case shown here, each block equilibrates in the globular-disordered conformational class (Chiariello et al., 2016).

Beyond the average trend of contact frequencies with genomic separation, the SBS model can explain other specific features of chromatin folding, such as the formation of Topological Domains (TADs, Dixon et al., 2012; Nora et al., 2012) and their higher-order hierarchical structure recently discovered (Fraser et al., 2015). To illustrate the basic concepts involved without delving into polymer physics details (see Nicodemi and Prisco, 2009; Barbieri et al., 2012, 2013; Chiariello et al., 2015, 2016; Annunziatella et al., 2016), we consider first a toy polymer of chromatin with two different types of beads and binders (block-copolymer model, Figure 3). The beads along the polymer are alternated in two pairs of blocks, which can interact only with the corresponding type of binders. Each polymer block can fold in different ways, according to thermodynamics phase it belongs to. The folding dynamics from the SAW initial configuration to the equilibrium state reveals some relevant aspects of chromatin architecture. First, consecutive globular structures, visually similar to the TADs, experimentally observed in Hi-C and GAM data, appear. Then, higher order structures appear, generating the typical hierarchical structure from interactions between the different blocks (Figure 3). In the toy model discussed here, the equilibrium contact matrix presents a chessboard-like pattern (Figure 3A), which reflects the hierarchical organization of higher-order structures, resulting from intra- and inter-domain interactions, even at lower energies. The example shown in Figure 3 refers to the case where an initially open polymer folds into the closed disordered state, as discussed before.

The simplified description of folding of the above toy model schematically illustrates the basic polymer mechanisms that could underlie the formation of complex patterns seen in the experimental contact data. All details from polymer physics, and the underlying theory, can be found in the cited original papers (e.g., Nicodemi and Prisco, 2009; Barbieri et al., 2012; Annunziatella et al., 2016; Chiariello et al., 2016).

## 3D genome reconstruction: the *Bmp7* locus in ESC-46C murine cells

**Figure 4:**
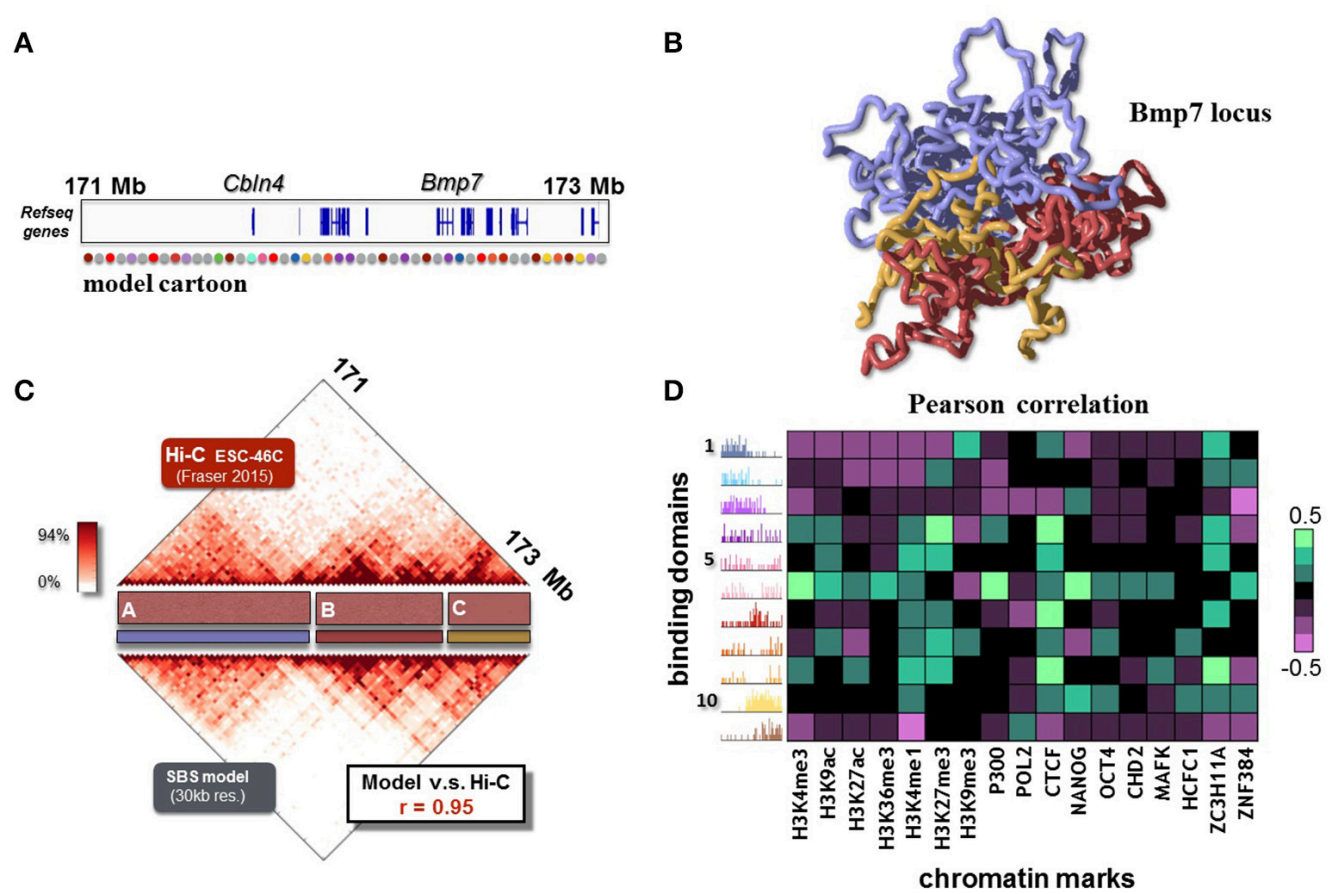
The folding of the *Bmp7* locus in ESC-46 cells is recapitulated by the SBS model. **(A)** The modeled 2.3 Mb region around *Bmp7* gene (chr2:171090000-173430000, mouse ESC-46C cells) is shown. A schematic representation of the SBS polymer model is reported in the bottom part. **(B)** 3D reconstruction from a Molecular Dynamics simulation of the *Bmp7* locus. It allows to visualize how TADs and higher-order metaTAD structures fold in space for this locus (adapted from Chiariello et al., 2016). **(C)** The SBS model recapitulates the experimental Hi-C data (binned at 30 Kb resolution, from Fraser et al., 2015) with good accuracy, as shown by the simulated contact map (Pearson correlation coefficient *r* = 0.95, adapted from Chiariello et al., 2016). **(D)** Pearson correlation coefficients between the relative abundance of binding domains with epigenetics chromatin features. The resulting barcode of the binding domains predicted by the SBS model links epigenetics to spatial architecture, and returns a potential map of the molecular nature of the factors driving folding.

After reviewing the general mechanisms of polymer physics, within the framework of the SBS model, that can explain chromatin folding, next we focus on the description of experimental contact data of specific loci (Annunziatella et al., 2016; Chiariello et al., 2016). Polymer modeling can, in fact, explain real data to a good degree of accuracy and return the full 3D structure of the loci of interest. As example, we briefly review the results about the *Bmp7* locus, a 2.3 Mb region around the *Bmp7* gene (chr2:171090000-173430000, Figure 4A), that is important for osteoblastic differentiation (Bandyopadhyay et al., 2006). The SBS model can reproduce the Hi-C data from ESC-46C murine cells (at 30 Kb resolution) with high accuracy (Pearson correlation coefficient *r* = 0.95, Figure 4B).

In order to explain the finer details of the locus experimental Hi-C matrix, the model is of course more complex than the two color toy polymer considered in the previous section. The *Bmp7* locus model requires 11 different types of binding sites (represented as colors in Figure 4C), and their corresponding binding molecules. The positions of binding sites on the polymer are estimated from the Hi-C data through a statistical inference algorithm and Monte-Carlo optimization (Annunziatella et al., 2016; Chiariello et al., 2016; Bianco et al., 2017). Interestingly, the inferred binding domains overlap roughly with the TADs visible in the Hi-C matrix. Nevertheless, they also compenetrate and partially overlap with each other, giving rise to the visible higher-order structures (Annunziatella et al., 2016; Chiariello et al., 2016). The 3D reconstruction of the model (Figure 4B) gives the 3D representation of the patterns contained in the Hi-C data. For example, the three principal domains of interactions labeled as A, B and C in Figure 4C, are clearly visible in the polymer snapshot (colored in medium purple, red and gold respectively). In SBS model these interactions naturally emerge because of the presence of molecules (binders) that mediate longer-range, higher-order interactions.

Finally, to give a sense of the molecular nature of the binding sites, we cross epigenomic databases of chromatin features with the relative abundance of the binding sites (Bianco et al., 2017). The heatmap of Figure 4D represents the Pearson correlation coefficients between the relative abundance of binding domains and chromatin features from ENCODE along the *Bmp7* region for ESC-46C cells. Each binding domain is characterized by specific combinations of epigenomic features: some domains, in fact, correlate with active marks, while others are characterized by more repressive marks. Interestingly many domains correlate strongly with CTCF, a known chromatin organizer, whilst others are not linked to it. That suggests, for instance, that while CTCF has an important role in the folding of chromatin (Tark-Dame et al., 2014), additional remodeling factors also play a role, beyond CTCF, as shown in recent studies (Barbieri et al., 2017).

## Application to neurogenetics: the *7q11.23* locus

As a final application of the model, we present here the first, albeit initial exploration of the 3D structure of the *7q11.23* locus, which is involved in severe neuropsychiatric disorders. Structural variants at the *7q11.23* locus can cause a variety of neurological, behavioral and other problems. For example, the *7q11.23* duplication syndrome is associated with speech problems and behavioral issues such as increased anxiety levels or autism (see Berg et al., 2007; Merla et al., 2010; Ramocki et al., 2010; Ebert et al., 2014). The Williams-Beuren syndrome (WSB) is a complex developmental disorder associated to the deletion of 1.5–1.8 Mb in the *7q11.23* locus, encompassing a couple of dozens genes (see Nature Research Highlights, 2011; Sanders et al., 2011; Chailangkarn et al., 2016; and ref.s therein).

The attribution of the various features of WBS to specific genes is a complex, on-going effort, relying, among other strategies, on the phenotype-genotype analysis of patients with atypical deletions, and on genetically modified mouse models. Besides the role of the genes in the deleted/duplicated region or epigenetic mechanisms, a factor that may be implicated in determining the genotype-phenotype relationship is the effect of deletions and duplication on the 3D architecture of the genomic region. For such a reason, we report a first investigation of the architecture of the locus.

**Figure 5:**
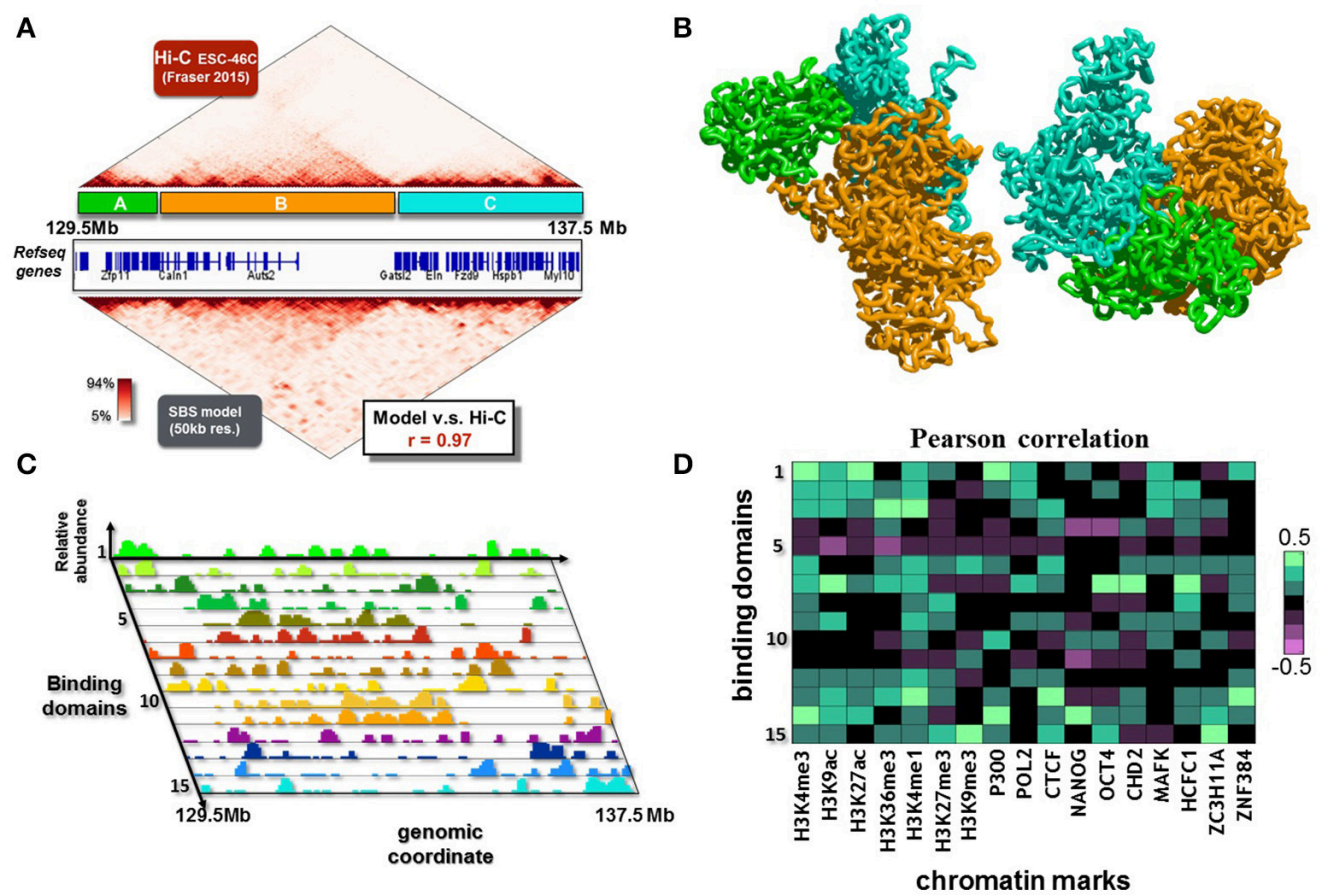
The 3D structure of the *7q11.23* locus in ESC-46C cells. **(A)** The modeled 8 Mb region (chr5:129500000-137500000, mouse ESC-46C cells) is shown. This region is syntenic with the *7q11.23* locus in human genome, which is linked to a variety of neurological disorders, such as autism or the Williams-Beuren syndrome. The Hi-C experimental data (binned at 50 Kb resolution, from (Fraser et al., 2015), top matrix) are reproduced by the contact matrix inferred from the SBS model (bottom matrix) with good accuracy (Pearson correlation coefficient *r* = 0.97). **(B)** Two independent 3D reconstructions derived by Molecular Dynamics simulations. The major contact domains of the locus have complex, long range interactions with each other (e.g., the contacts between the green and cyan domains), in agreement with the Hi-C data. **(C)** The SBS inferred binding domains of the locus, which drive its folding, are here shown. On the z-axis, their relative abundance (each binding domain is represented with a different color, on the y-axis) is shown as a function of the genomic coordinate (x-axis). **(D)** Pearson correlation coefficients between the relative abundance of binding domains with epigenetic chromatin features.

Here, we consider a 8 Mb region (chr5:129500000-137500000, Figure 5A) from mouse ESC-46C cell line, syntenic with the *7q11.23* locus in human genome. The dataset used is from Fraser et al. (2015), binned at 50 kb. The inferred contact matrix is highly correlated with the experimental matrix (Figure 5A), with a Pearson correlation coefficient *r* = 0.97. The polymer model of the locus involves 15 different types of binding sites, whose position and abundance along the DNA sequence is represented by the different color histograms in Figure 5C. The associated inferred conformations of the polymer model help explaining the 3D features of this locus. In Figure 5B, two possible configurations, obtained from independent simulations, are showed. For sake of clarity, we color in green, orange and cyan respectively the three major domains visible in the matrix (labeled again as A, B and C, Figure 5A), so we can easily compare the contact pattern with the spatial reconstruction. At a first visual analysis, we can recognize the A, B and C domains as distinguishable and individual blocks, in agreement with the experimental data. Interestingly, a more deeper inspection reveal a non-random contact between domain A and domain C, which is again in agreement with the long range interaction contained in the experimental matrix, even though it is an higher-order detail. Importantly, a collection of much smaller domains (the strong red triangles in the contact matrix) close to the diagonal is evident, and a complex pattern of higher order interactions among them is present, so to give the typical hierarchical internal substructure to the major domains. The model also captures such lower level organization. For sake of clarity, we do not color all these domains in the polymer representation of Figure 5B. As the model does capture not only general aspects of the locus organization, but also its finer features, it can be used to derive relevant biological implications of the 3D structure.

To this aim, we analyse epigenomic databases of chromatin features and cross them with the relative abundance of the binding sites (Bianco et al., 2017), as made in the previous section. In Figure 5D are reported the results, which reveal a complex, not trivial, pattern of correlations. Interestingly, several binding domains exhibit high correlations with many of the considered features, reflecting the biological complexity of the locus, highly enriched in genes (Figure 5A). Conversely, few of the binding domains (e.g., type 5 and 11, Figures 5C,D) do not correlate with the considered epigenomic features, and result to be associated with the central, gene poor, region of the locus.In summary, our polymer model of the murine genomic region syntenic with the *7q11.23* locus in human provides a first reconstruction of the ensemble of 3D conformation of the locus. In particular, a complex network of higher-order interactions of the locus emerges from our analysis, whose rewiring could be important to understand the effects of disease associated structural variants.

## Discussion

In conclusion, we reviewed recent developments in polymer physics models to understand the 3D structure of genomic loci and the connection to human diseases. In particular, we discussed a polymer model of chromatin, the SBS model, where folding is determined by specific interaction with DNA-binding sites. Genome-wide and loci specific chromatin contact data can be explained over orders of magnitude in genomic separation, and also other chromatin features as TADs (Dixon et al., 2012; Nora et al., 2012) and spontaneous hierarchical domains formation (Fraser et al., 2015). As mentioned in the Introduction, other polymer models, have been proposed. These models also reproduce successfully experimental data for different genomic regions. For instance, in the model proposed in Brackley et al. (2013), the α and β globin gene regions in mouse erythroblasts are explained with a high degree of accuracy (Brackley et al., 2016), while the loop extrusion model reproduces the contact data of loci where CTCF factor is known to play an important role (Sanborn et al., 2015; Fudenberg et al., 2016). Another important example is the *Xist* locus in mouse embryonic stem cells (Scialdone et al., 2011; Giorgietti et al., 2014; Chiariello et al., 2016). Non mammalian genomes have also been modeled, as *Drosophila* (Jost et al., 2014) and budding yeast (Koziol et al., 2005; Cheng et al., 2015).

The SBS is, of course, a simplified polymer model of chromatin and many other factors are present in real cells, as confinement, crowding and entanglement effects. Additionally, off-equilibrium phenomena are likely to be present as well-known from the studies of complex fluids (see Caglioti et al., 1998; Nicodemi, 1998; Nicodemi and Coniglio, 1998; Coniglio and Nicodemi, 2000; Nicodemi and Jensen, 2001; Tarzia et al., 2004; Nicodemi and Prisco, 2007; Grebenkov et al., 2008, and references therein). The SBS has been also used to describe symmetry-break mechanisms in the *Xist* region during X-Chromosome Inactivation (Nicodemi et al., 2008), and to model the chromosome recognition and pairing in mitosis and meiosis (Ong and Corces, 2014). The SBS model has been employed to explain folding of a set of important loci, such as the *Hoxb* locus (Annunziatella et al., 2016; Barbieri et al., 2017) and the *Sox9* locus (Chiariello et al., 2016). Here, we illustrated the case of the the *Bmp7* locus in ESC-46C murine cells (Chiariello et al., 2016).

As a novel application of potential interest to neurogenetics, we presented the reconstruction of the *7q11.23* locus architecture, where structural variants (deletions and duplications) are known to be linked to neurocognitive disorders such as autism spectrum disorders or the Williams–Beuren syndrome (WBS). For example, a clear genotype–phenotype correlation has been determined in WBS only for some genes (e.g., the elastin gene, which give rise to the vascular and connective tissue abnormalities). The molecular substrates carrying the other clinical aspects of *7q11.23* copy number variations (CNVs), including the neurocognitive phenotypes, are still not fully understood. Recent studies suggest that other factors, as regulatory sequences or epigenetic mechanisms, could have an important role in producing the variable expressivity of *7q11.23* CNV phenotypes, besides the role of the genes in the deleted/duplicated interval. Understanding the folding mechanism that regulates the 3D conformation of this region can also help a better comprehension of the link between the structural variants and the rewiring of the contacts between the locus genes and their regulators. To that aim, we employed our polymer physics model. We showed that a complex pattern of interaction in this large genomic region is present. Investigating the spatial structure of this locus, and analogously of loci whose variants are involved in diseases, can help to better understand the contact landscape between their regulatory elements. In this way, the effect of structural variants on the spatial organization can be quantitatively studied, and the comprehension of the mechanisms causing the disease can be potentially widely improved.

Importantly, in our model it is not required additional *a priori* information about the molecular factors shaping the genome (for instance, CTCF sites position and other epigenetics features). Furthermore, it can be used also for modeling non mammalian genomes, whereas contact data are available, as in yeast (Hsieh et al., 2015). The putative responsible factors are purely derived by experimental contact data and their nature guessed by correlating their position with epigenomics datasets. That can be important to novel applications in biomedicine to diagnose *in-silico* diseases associated to improper chromatin folding, as cancer (Valton and Dekker, 2016) and congenital disorders (Ong and Corces, 2014; Lupiáñez et al., 2015).

## Materials and methods

We investigated the SBS polymer model by Brownian Molecular Dynamics simulations, implemented using LAMMPS (Plimpton, 1995). The complete description of methods and details about the model are provided in the cited references.

The data files of chromatin features in the *Bmp7* and *7q11.23* regions in mouse ESC used here are available online from ENCODE (The ENCODE Project Consortium, 2012), with the accession numbers: ENCFF434DJA, ENCFF916EVG, ENCFF945LRL, ENCFF001XWR, ENCFF001YIX, ENCFF001YAD, ENCFF251JZH, ENCFF817CZF, ENCFF001XWO, ENCFF001YIY, ENCFF001YIV, ENCFF830QDG, ENCFF001YAE, ENCFF796LDS, ENCFF883ROD, ENCFF530LPO, ENCFF854IVF.

## Author contributions

MN designed the study; CA, AE, SB, AC, and MN developed the project; AC, CA, SB, AE, and LF run the computer simulations and performed the analyses; AC, CA, AE, SB, AP, and MN wrote the manuscript.

### Conflict of interest statement

The authors declare that the research was conducted in the absence of any commercial or financial relationships that could be construed as a potential conflict of interest.
